# Infrared retinal images for flashless detection of macular edema

**DOI:** 10.1038/s41598-020-71010-0

**Published:** 2020-09-01

**Authors:** Aqsa Ajaz, Dinesh K. Kumar

**Affiliations:** School of Engineering, RMIT University, Melbourne, Australia

**Keywords:** Biomarkers, Signs and symptoms

## Abstract

This study evaluates the use of infrared (IR) images of the retina, obtained without flashes of light, for machine-based detection of macular oedema (ME). A total of 41 images of 21 subjects, here with 23 cases and 18 controls, were studied. Histogram and gray-level co-occurrence matrix (GLCM) parameters were extracted from the IR retinal images. The diagnostic performance of the histogram and GLCM parameters was calculated in hindsight based on the known labels of each image. The results from the one-way ANOVA indicated there was a significant difference between ME eyes and the controls when using GLCM features, with the correlation feature having the highest area under the curve (AUC) (A_Z_) value. The performance of the proposed method was also evaluated using a support vector machine (SVM) classifier that gave sensitivity and specificity of 100%. This research shows that the texture of the IR images of the retina has a significant difference between ME eyes and the controls and that it can be considered for machine-based detection of ME without requiring flashes of light.

## Introduction

Macular edema (ME) refers to swelling within the retinal tissues that occurs when damaged blood vessels leak fluid and protein deposits into the macula region, leading to tissue thickening and distorting vision^1^. ME is irreversible and is the major cause of a decrease in visual acuity in patients with diabetes^2^. Early diagnosis and monitoring of ME can decrease the risk of vision loss.

The diagnosis and monitoring of ME require retinal imaging; here, the three routinely used modalities are as follows: colour fundus photography (FP), fluorescein angiography (FA) and optical coherence tomography (OCT). Some of the recent advancements in the field include the use of hyperspectral imaging and infrared imaging^3^. Various automatic methods for ME detection and grading using image processing and pattern recognition techniques have been investigated^3–39^. Previously, a number of methods have been proposed for grading diabetic macular oedema (DME) based on the location and segmentation of exudates^13,25,38^ and macula or on the extraction of texture or image-based features^23,40^.

A texture analysis is performed by extracting the statistical feature sets from the local distributions, which can be used later for segmentation or classification purposes. The gray-level co-occurrence matrix (GLCM) for obtaining the texture features were introduced by Haralick in 1973, and this has been widely used in retinal image analyses^41,42^. Lim et al. used a modified combined local binary pattern to extract local gray-level features of all channels and then a support vector machine (SVM) classifier to classify DME. The proposed method yields a sensitivity and specificity of 80% and 70%, respectively^20^. Jerald et al. extracted global features such as intensity, colour and texture for detecting the severity of DME. Hard exudates were detected using an extreme learning machine classifier (ELM); the detection performance had an accuracy rate of 98%, sensitivity of 99.5% and specificity of 85–98%^43^. Tariq et al. used morphological features and a Gabor filter to segment the exudates; then, the distance between the exudate and macula centre was used to grade the severity of DME^44^.

One common limitation when it comes to retinal vasculature examinations is that an eye-fundus examination requires a flash of light, which is unpleasant and causes short-term blindness for most people; however, a small number of people are intolerant to this. OCT provides a cross-section of the retina that is suitable for detecting ME but has limited availability in remote regions^45–47^. One option is to use the IR image of the retina, which does not require a flash of light and is routinely performed during the step before OCT. Infrared imaging offers certain advantages over the traditional colour FP. The ocular fundus shows a high reflection of IR compared with visible light and has a longer depth of penetration that can reach into retinal sublayers. Compared with color images, IR produces a better vessel to background contrast and is suitable for detecting subretinal pathologies. Moreover, IR images improve the quality of illumination by removing the out-of-focus, scattered components of the reflected light^48,49^. Further, IR imaging does not require a flash of light and, hence, is less traumatising. These images have the potential to provide deeper visualisation of the retina, including the choroidal vessels, because it comprises longer wavelengths compared with the Green channel, which is commonly used in colour FP. Thus, detecting pathologies, even in the presence of haemorrhages and cataracts, which may go undetected under other imaging systems^50–52^.

IR imaging is routinely used during an OCT examination to view the structure of the retina, subretinal lesions and accumulation of fluid in the retina; to image patients with choroidal neovascularization^53–56^, with age-related macular degeneration^57,58^ and with Stargardt’s disease;^59^ and to provide information about the site of leakage and leakage patterns. However, the use of IR images for macular edema has not been reported. There is also no reported GLCM analysis of IR images of the retina.

The current paper reports the differences between the IR images of the retina of eyes with ME and eyes without ME. To overcome the limitation of poor contrast and for the unsupervised analysis of these images, global features of the image were investigated.

## Results

The performance of the proposed method was evaluated on a dataset of 41 IR images, which are described in the methodology section. The dataset consists of 18 eyes of control subjects who had no sign of Diabetic Retinopathy (DR) or DME and 23 eyes with clinically diagnosed ME.

Histogram and GLCM parameters were extracted from IR images of ME eyes and controls. Statistical analyses were performed using MedCalc 10.0.2.0 (MedCalc Software Ostend, Belgium) for both the histogram and GLCM parameters. The statistical distribution was obtained and evaluated using the Shapiro–Wilk test. A one-way ANOVA was performed to determine statistically significant group differences between the control and ME cases. Table 1 shows the comparison of the texture parameters obtained from IR images of the control and ME cases.

**Table 1:** Comparison of the texture parameters between IR images of the control and ME cases.

	Parameter	Control	ME cases	P-value*
Histogram	Mean	4.68	4.53	0.07
	Skewness	− 0.20	− 0.05	0.08
	Kurtosis	3.45	3.42	0.84
	Entropy	0.257	0.25	0.75
	Variance	1.58	1.44	0.15
	Energy	1.57	1.56	0.81
GLCM	Autocorrelation	15.09	16.61	0.04*
	Contrast	0.143	0.107	0.007*
	Correlation	0.997	0.961	< 0.001*
	Cluster shade	− 4.09	− 2.15	0.19
	Cluster prominence	107.19	111.0	0.80
	Dissimilarity	0.125	0.101	0.03*
	Homogeneity	0.939	0.950	0.04*
	Diffuse variance	0.143	0.107	0.007*
	Diffuse entropy	0.378	0.325	0.02*
	The infinite measure of correlation 2	0.933	0.948	0.001*
	Sum average	7.04	7.73	0.06
	Sum entropy	1.83	1.83	0.96
	Sum variance	36.95	40.05	0.04*
	Maximum probability	0.38	0.33	0.86
	Inverse difference moment normalised (IDMNC)	0.997	0.998	0.008*
	Inverse difference normalised (INDNC)	0.986	0.988	0.04*

*p-value from a one-way ANOVA.

The six histogram parameters that do not show statistically significant differences between the case and control are as follows: the mean, skewness, variance, kurtosis, entropy and energy (p > 0.05). Among the GLCM parameters the features autocorrelation, contrast, correlation, dissimilarity, homogeneity, diffuse variance, diffuse entropy, sum variance and inverse difference moment normalised are the parameters that show a significant difference between the cases and controls. Other GLCM features were not found to be significantly different between the two groups.

In the current work, an SVM classifier was used for classifying the features of the IR images, and a “leave-one-out” cross-validation method was used to validate the results. In this method, the learning algorithm can be tested once for each instance after it is trained using all the other instances of the dataset^60^. The results show a sensitivity, specificity and accuracy of 100% when using an SVM classifier with five top-ranked texture features of IR images.

The diagnostic performance for diagnosing ME was calculated using the cut-off values for each GLCM parameter according to the Youden Index. The receiver operating characteristic (ROC) was constructed, and the area under the curve (AUC), here referred to as A_Z_, for each parameter was calculated. The ROC curve provides sensitivity versus specificity, while the AUC estimates the overall performance.

**Figure 1:**
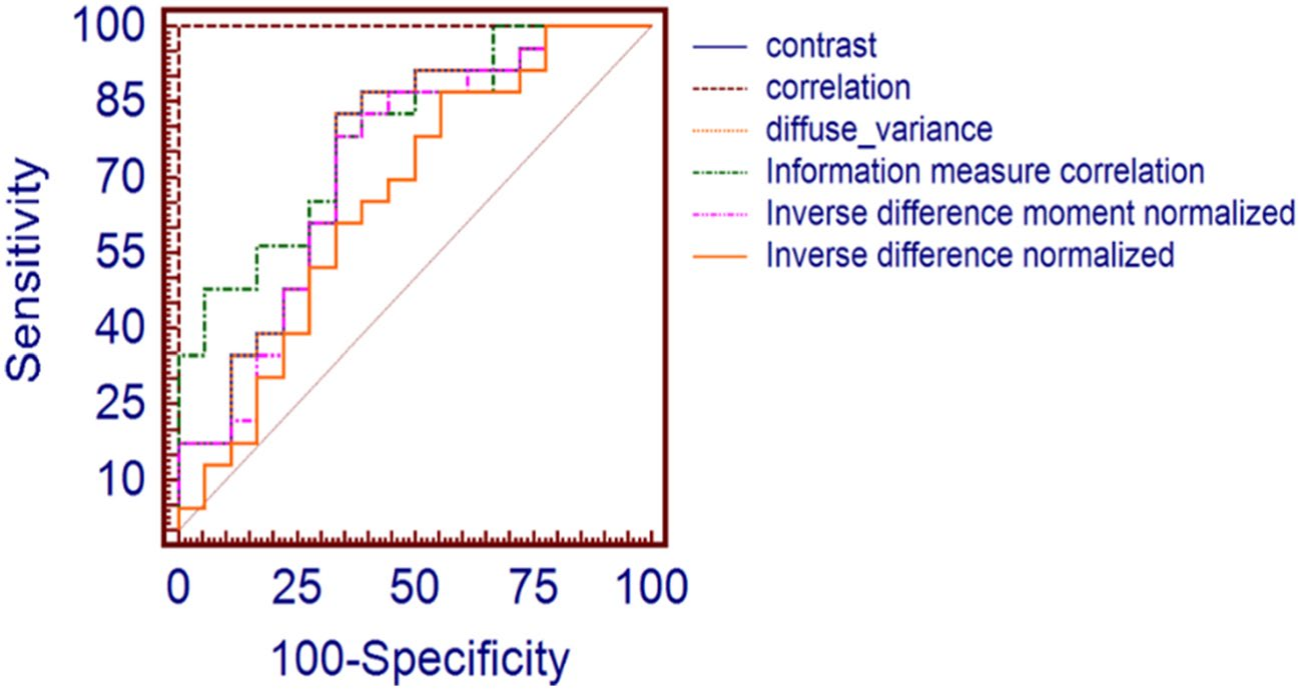
The ROC curve analysis for the top six GLCM features used for categorising ME. Among these GLCM parameters, the correlation feature has the highest AUC (A_Z_) value; A_Z_ = 1 and is the most suitable for differentiating between the ME case and control subjects.

The diagnostic performance using the cut-off values were calculated for each significant GLCM parameter to diagnose ME; these are summarised in Table 2. Among these GLCM parameters, the correlation feature has the highest AUC (A_Z_) value; A_Z_ = 1, having a sensitivity, specificity and accuracy of 100%. Figure 1 shows the ROC curve and AUC for the top six GLCM parameters for categorising ME.

**Table 2:** Diagnostic performance of the GLCM features for detecting ME.

	Parameter	Cut-off	Sensitivity	Specificity	AUC (A_z_)
GLCM parameters	Contrast	≤ 0.13	82.61	66.67	0.74
	Correlation	≤ 0.98	100	100	1.00
	Diffuse entropy	< 0.38	86.96	50.0	0.69
	Diffuse variance	≤ 0.13	82.61	66.67	0.74
	Dissimilarity	≤ 0.12	86.96	50.0	0.67
	Homogeneity	> 0.93	86.96	44.4	0.64
	Inverse difference moment normalised	> 0.99	78.26	66.67	0.72
	Inverse difference normalised	≤ 0.98	86.96	44.44	0.65
	The infinite measure of correlation 1	> 0.72	78.26	66.67	0.78
	The infinite measure of correlation 2	> 0.93	86.96	72.22	0.78

**Figure 2:**
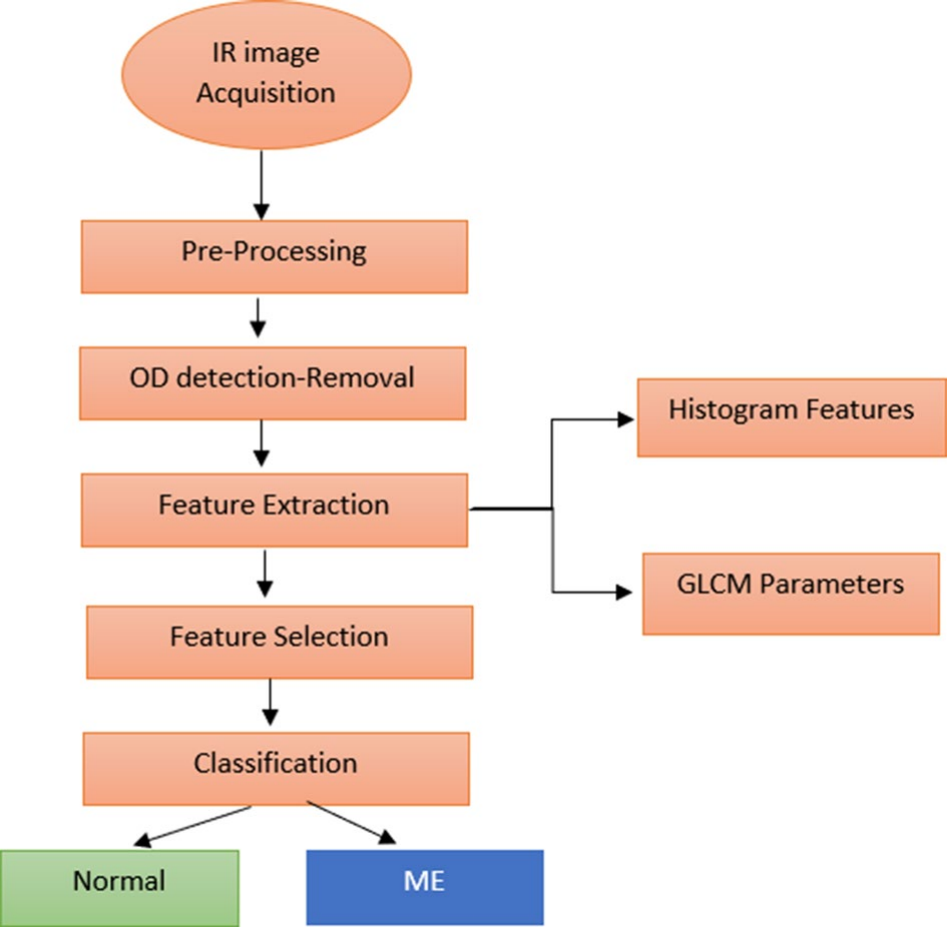
The framework of the proposed method for the detection and classification of ME cases and controls.

## Discussion

The current research proposes the use of an IR image of the retina as an alternative modality for detecting ME. IR imaging has the advantage that it does not require a flash of light or dilation of the pupil and can be performed by inexpensive eye-fundus imaging.

Several eye-examination devices such as OCT incorporate the use of infrared images to support the scan. Although IR has number of potential advantages, it suffers from some technical limitations. Some of these include the following: (a) the presence of hyperreflective artefacts—related to reflection or light-scatter because of posterior chamber intraocular lenses—in almost 25% of eyes, here most commonly in pseudophakic patients^61,62^, and (b) restricting the illumination wavelength to an IR band that emphasises the subretinal structures at the expense of other layers^63,64^. IR reflectance images also lack direct quantitative measures of retinal thickness. Finally, IR images have low contrast, blurred edges with a central light reflex that causes a light streak along the vessel length, making the segmentation of these images a challenging task. Our previous work overcame some of these limitations by enhancing the quality of the image and segmentation of retinal vasculature using a series of morphological operations^65^. Our work has as shown that for healthy eyes, the vasculature information in IR images is comparable with the colour fundus images.

A texture analysis gives global measures of the texture of the image and has been used for medical images to identify disease conditions. It has the advantage of not requiring segmentation of the images, and it can be performed automatically and without supervision. In the current paper, we have proposed the automatic detection of ME using first- and second-order texture features of IR retinal images, identifying the most significant features that can be used for differentiating between ME eyes and eyes with no ME or DME.

The results from the one-way ANOVA test show that there is no statistically significant difference between healthy eyes and ME eyes for the histogram features, with all *p*-values > 0.05. However, 10 Haralick texture features—autocorrelation, contrast, correlation, dissimilarity, homogeneity, diffuse variance, diffuse entropy, an infinite measure of correlation, sum variance, inverse difference and inverse difference moment normalised—extracted using the GLCM matrix showed a statistically significant difference between the controls and ME cases; *p*-value < 0.05.

Feature selection was performed using the ANOVA filter-based method, which selects the top-ranked features as an input to the classifier. The performance of the proposed method was evaluated based on the ROC curve. The results show that the GLCM parameter ‘correlation’ is the most suitable for differentiating between the ME case and control subjects, with AUC = 1.0, here having 100% sensitivity and specificity. A comparison of the proposed method with several previous works reported in the literature is shown in Table 3; this shows that the method described in the current paper is better than the other methods. Another potential advantage of this method is that it uses IR retinal images, which have been reported to detect pathologies even in presence of haemorrhages and cataracts, which can go undetected when using other imaging systems^51,52^. This is also the first time the GLCM of IR images have been reported.

**Table 3:** Summary of various methods used for automatic detection of ME using colour FP, FA and OCT.

Author	Imaging type	Database	Method and classifiers	Performance index
Nayak et al.^25^	Colour fundus photography	Private (350)	Matched correlation and neural network	Sensitivity—95.40% Specificity—100%
Siddalingaswamy et al.^34^		Private (148)	Clustering and location of exudates	Sensitivity—95.60% Specificity—96.15%
Fleming et al.^12^		Private (14,406)	Morphological image processing, exudate location	Accuracy—99.2% (NCSME) Accuracy—97.3% (CSME)
Lim et al.^20^		MESSIDOR (88)	Watershed transform and exudate location	Sensitivity—80.90% Specificity—90.20% Accuracy—85.20%
Ang et al.^6^		Private (90)	Mathematical morphology and neural network	Sensitivity—90% Specificity—100% Accuracy—96.67%
Akram et al.^4^		MESSIDOR (1,200)	Morphological image processing features extracted from filter bank response, energy and support vector machine	Sensitivity—92.60% Specificity—97.80% Accuracy—97.30%
Giancardo et al.^13^		HEI-MED and MESSIDOR (1,200)	Wavelet transform, Kirsch edge detection, colour, and support vector machine	AUC—0.94
Punnolil et al.^28^		DRIVE, DIARETDB1, STARE (251)	Morphological features of exudates, texture and SVM	Sensitivity—96.89% Specificity—97.15%
Alipour et al.^5^		Private (75)	Curvelet and foveal avascular zone (FAZ) size	Sensitivity—93% Specificity—86%
Tariq et al.^44^		MESSIDOR and STARE (1,281)	Gabor filter, thresholding and support vector machine	Accuracy—97.20% (MESSIDOR) Accuracy—97.53% (STARE)
Tariq et al.^38^		MESSIDOR and STARE (1,281)	Morphological features of exudates, Gabor filter, thresholding, texture and Gaussian mixture model	Accuracy—97.30% (MESSIDOR) Accuracy—97.89% (STARE)
Medhi and Dandapat^22^		DRIVE, DIARETDB1, and HRF (174)	Top hat filtering, thresholding and exudate location	Sensitivity—97.5% Specificity—98.7%
Ibrahim et al.^18^		Private (300)	Entropies, fuzzy Sugeno, discrete wavelet transform, and neuro-fuzzy interference	Accuracy—95.93% (MESSIDOR) Accuracy—98.55%
Aditya et al.^19^		MESSIDOR (1,200)	Texture features	Sensitivity—91% Specificity—75% Accuracy—80%
Rabbani et al.^29^	oct, fa, fp oct	24 eyes	Segmentation of leakage areas in FA	Active contour model, accuracy—86.6%
Goebel et al.^14^		136 eyes	OCT can detect macular oedema with great reliability; retinal thickness correlated with FA leakage in angiograms	The sensitivity of the system for detecting CSME was 89% with a specificity of 96%
Yang et al.^39^		33 eyes	OCT showed a mean standard deviation foveal thickness as 255.6 ± 138.9 μm in CSME eyes and 174.6 ± 38.2 μm in eyes without CSME (p = 0.051)	
Arif et al.^7^		62 eyes	The discriminant analyser was used to classify retina oedema using OCT	Accuracy 100%—retinal oedema patients, 91.8%—healthy
Bilal et al.^15^		90 oct volumes	Detection and grading of maculopathy using coherent tensor features from OCT volume and 7D vector features (three features—retinal thickness profile and four features—retinal fluids)	Accuracy—97.98%
Sugmuk et al.^36^		16 images	RNFL segmentation to find the drusen and then the classification of disease into Age Related Macular Degeneration (AMD) and DME using the binary classifier	
Pai et al.^26^		3 images	OCT shows some volcano signs in the vitreo-foveolar interface in patients' chronic DME	
Sadda et al.^30^		71 eyes	Grid scanning OCT was used for the detection of CSME	System sensitivity 89% and specificity 85%
Schaudig et al.^33^		22 patients	A significant difference in retinal thickness was found between the subjects having diabetic retinopathy and normal	
Tocino et al.^32^		111 subjects	Foveal thickness was a strong and independent predictor of CSME	AUC for this predictor-0.92; for a cut-off point of 180 micron, the sensitivity was 93% and specificity 75%
Syed et al.^37^		90 OCT volumes	Automatic diagnostic of ME and central serous retinopathy using 3D retinal surface	Accuracy—98.88% Sensitivity—100% Specificity—96.66%
Martinez et al.^21^		277 eyes	Detection and validation of OCT using foveal thickness and intraretinal fluid Binary logistic regression model	Accuracy—0.88 Sensitivity—0.83 Specificity—0.89
Panozzo et al.^27^		1,200 eyes	Classification of ME using OCT The classification takes into account five parameters: retinal thickness, diffusion, volume, morphology and presence of vitreous traction for determine the severity of ME	
Hassan et al.^16^		71 images	Segmentation of retinal layers using OCT Coherent tensor used	SVM classifier, Accuracy—97.78%
Dash et al.^11^		55 IMAGES	Pattern classification techniques	Sensitivity—95% Specificity—100% Accuracy—96%
Samagaio et al.^31^			Multi-level image thresholding approach	F-Measures of 87.54% and 91.99% for the Diffuse Retinal Thickening (DRT) and Cystoid Macular Edema (CME) detections, respectively
Sibide et al.^35^		Two datasets: 32 SD-OCT and 45 SD-OCT volumes	Anomaly detection for DME detection	Sensitivity and a Specificity of 80% and 93% on the first dataset, and 100% and 80% on the second one
Our proposed work		Private (44)	Histogram and GLCM texture features, SVM, KNN and Naïve Bayes	KNN and SVM: Sensitivity—100% Accuracy—100% Naïve Bayes: Sensitivity—100% Accuracy—97.6%

One of the limitations of the present study is that the sample size is small, and the study is only cross-sectional. Longitudinal studies with a larger number of patients are necessary to validate the results before these can be considered to be used in clinical practice.

Deep learning algorithms have shown a high level of performance for the classification of medical images and have been developed for the detection of DR and DME^66^. In the future, integrating deep learning algorithms for extracting features, segmentation and classification could help in the automatic detection of ME when using IR retinal images.

## Methodology

### Data collection

The current study investigated the IR images of the retina of ME patients who presented at Gladstone Park Eye Clinic, Melbourne, Australia, irrespective of any aetiologies such as diabetes, central retinal branch vein occlusion and dye leakage associated with choroidal neovascularisation syndrome. The study was approved by the RMIT human ethics committee and conducted following Helsinki accord 1986 (modified 2004). The experimental protocol was explained in plain language to each participant, and written informed consent was obtained before the experiment. An optic disc-centred IR image was obtained from each participant using the Spectralis SD-OCT (Heidelberg Engineering, Heidelberg, Germany) with an integrated IR-SLO imaging system, having a λ = 830 nm, FOV = 30 × 30 degree and 768 × 768 pixels minimum image size. A total of 41 images of 21 subjects—23 cases and 18 control images—were used. All the volunteers (controls) self-declared themselves as healthy, non-smokers, moderately active and with no history of diabetes, hypertension or retinopathy. Two experienced clinicians visually inspected the OCT B-scans for structural changes, such as the presence of intraretinal cysts, thickened posterior vitreous surface adhering to the macula, sponge-like retinal swelling, cystoid macular oedema and serous retinal detachment^67^, and graded the IR images as ME present (cases).

### Methods

The current paper presents an automatic method for the detection of ME using the first- and second-order texture features of IR retinal images. The proposed system is accomplished in four stages: (i) image pre-processing, (ii) feature extraction, (iii) feature selection and (iv) classification. The framework of the proposed method is shown in Fig. 2.

#### Image pre-processing

IR images suffer from noise and low contrast, making pre-processing of these images a crucial step. This improves the quality of the image by reducing the noise and uneven illumination in the images and enhancing the contrast. A two-step approach was used for this purpose: IR retinal images were first filtered using a median filter, and this was followed by a contrast enhancement procedure using contrast-limited histogram equalisation (CLAHE).

**Figure 3:**
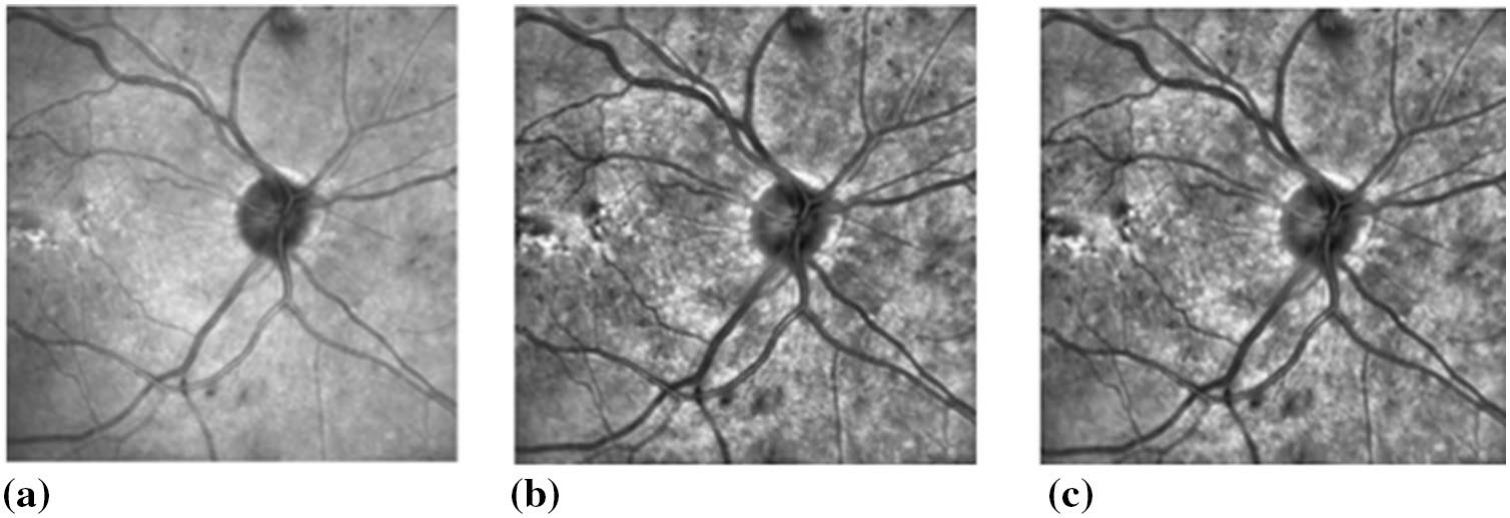
An example of pre-processing operations performed on the IR retinal image. (**a**) Original IR image. (**b**) IR image after performing CLAHE. (**c**) Median filtered IR image.

A median filter is a nonlinear filter with edge-preserving properties that reduce noise without compromising the edges. This was used to remove the Gaussian and Speckle noise^68^. Contrast enhancement was performed by using CLAHE with regional operations and suitable retinal images, which may have light intensity variations across the image^69^. Figure 3 shows an example of the pre-processing performed on IR retinal images prior to feature extraction.

The optic disc (OD) appears as a bright, yellow region with a higher colour intensity than the surrounding retinal areas. In the current study, for the automatic detection of macular oedema, we focused on the texture of the exudates, microaneurysm and blood vessels in the IR retinal image. To reduce the effect of intensity variations caused by optic disc, segmentation of OD was performed by pre-processing using contrast stretching, CLAHE and morphological opening and closing operations^70^.

#### Feature extraction

Feature extraction is an important step in designing an automatic diagnostic system^71^. Statistical texture features have been reported as useful for the classification of retinal images by analysing the spatial distribution of the gray levels, computing the local features and obtaining a statistical distribution of the local features.

Statistical texture analysis methods are classified as first-, second- and higher-order based on the number of pixels that define the local features. In first-order statistics, only one pixel is involved, and a pair of pixels are used for the second-order statistics^72^.

In the current study, we investigated the first- and second-order texture features, that is, histogram and GLCM features for the extraction of a texture from IR retinal images for classifying these images to detect ME cases. This was performed after these images had been pre-processed, as described earlier.

### First-order statistics—histogram texture features

The first-order statistics histogram texture features provide a 1D histogram of the image based on its gray-level values. The histogram of an image gives a count of how many pixels an image possesses with a given gray-level value. The probable density (*p*(*i*)) of occurrence of intensity level is calculated by dividing the values *h*(*i*) by the total number of pixels in *the N*_*x*_ *×* *N*_*y*_ image^73^, and *h*(*i*) represents the intensity level histogram .

1 $$ p(i) = \frac{h(i)}{{N_{x} N_{y} }} $$

We considered the gray levels in the image range from *0* *≤* *i* *≤* *N*_*g*_ *−* 1, where *N*_*g*_ is a total number of particular gray levels.

A histogram describes the characteristics of an image, for example, a narrowly distributed histogram represents a low contrast image^74^. The features extracted from a histogram that can be used to characterise textures are called central moments. The most commonly used central moments are mean, variance, kurtosis, energy, entropy and skewness. Mean defines the average level of intensity in an image. The variance describes the variation of intensity around the mean. Skewness is the measure of the asymmetry of gray-level values around the mean. Kurtosis gives a measure of the flatness of the histogram. While, energy gives an estimate of the uniformity of the intensity level distribution, entropy is a measure of randomness or degree of disorder present in an image. The entropy value is the largest when all the elements of the co-occurrence matrix are the same and small when the elements are unequal^75^. A simple image has a low entropy, while a complex image entropy value is high^76^.

### GLCM features

The GLCM is a statistical method for extracting second-order statistical texture features from an image. It characterizes the texture of an image by calculating how often a pair of pixels with a specific value and relationships occur in an image. The GLCM is a square matrix (G) with dimension *N*_*g*,_ where *N*_*g*_ is the number of gray levels in the image.

[*i, j*] represents the number of times a pixel value *i* is adjacent to pixel value *j* in an image and then dividing the entire matrix *i* by the total number of such comparisons made. Each entry in the matrix represents the probability of pixel value *i* to be found adjacent to the pixel value *j*^77^.

2 $$ G = \left[ {\begin{array}{*{20}c} {p(1,1)} & {p(1,2)} & {p(1,N_{g} )} \\ {p(2,1)} & {p(2,2)} & {p(2,N_{g} )} \\ {p(Ng,1)} & {p(N_{g} ,2)} & {p(N_{g} ,N_{g} )} \\ \end{array} } \right] $$

**Figure 4:**
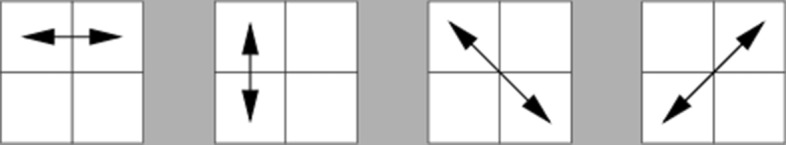
The four directions of adjacency used to calculate the Haralick features. The Haralick statistics are generated for co-occurrence matrix using these directions.

Because the adjacency can be defined to occur in each of four directions (horizontal, vertical, left and right diagonals) in 2D, for a square pixel image for four matrices can be calculated. Figure 4 shows the four directions of adjacency used to calculate the Haralick texture features^77^.

Haralick et al.^78^ proposed a method for using the GLCM to quantify the spatial relationship between neighbourhood pixels in an image. Haralick features have been successfully used in various application for the analysis of skin cancer and medical image analysis^41,42,79–82^. In the current paper, we have extracted the texture features from the probability matrix to classify macular oedema from IR retinal images. Around 56 GLCM parameters that include 14 Haralick features were extracted in four directions 0°, 45°, 90° and 135° using the IR images^78,83–85^. No other study has investigated the GLCM features of IR images. Table 4 shows the important Haralick features calculated from the IR retinal images. Haralick texture features were computed using these equations and the notations mentioned below.

**Table 4:** Haralick texture features calculated from the GLCM matrix.

Autocorrelation^84^		$\mathop \sum \limits_{i = 1}^{{N_{g} }} \mathop \sum \limits_{j = 1}^{{N_{g} }} \left( {i.j} \right)p\left( {i,j} \right)$
Contrast^78^		$\mathop \sum \limits_{i = 1}^{{N_{g} }} \mathop \sum \limits_{j = 1}^{{N_{g} }} \left( {i - j} \right)^{2} p\left( {i,j} \right)$
Correlation^78^		$\mathop \sum \limits_{i = 1}^{{N_{g} }} \mathop \sum \limits_{j = 1}^{{N_{g} }} \left( {\frac{{i - \mu_{x} }}{{\sigma_{x} }}} \right)\left( {\frac{{j - \mu_{y} }}{{\sigma_{y} }}} \right)p\left( {i,j} \right)$
Cluster prominence^78^		$\mathop \sum \limits_{i = 1}^{{N_{g} }} \mathop \sum \limits_{j = 1}^{{N_{g} }} \left( {i + j - 2\mu } \right)^{3} p\left( {i,j} \right)$
Cluster shade^78^		$\mathop \sum \limits_{i = 1}^{{N_{g} }} \mathop \sum \limits_{j = 1}^{{N_{g} }} \left( {i + j - 2\mu } \right)^{4} p\left( {i,j} \right)$
Difference entropy^78^		$- \mathop \sum \limits_{i = 1}^{{N_{g} - 1}} p_{x - y} \left( k \right)\log p_{x - y} \left( k \right)$
Difference variance^78^		$\mathop \sum \limits_{i = 1}^{{N_{g} - 1}} \left( {k - \mu_{x - y} } \right)^{2} p_{x - y} \left( k \right)$
Dissimilarity^84^		$\mathop \sum \limits_{i = 1}^{{N_{g} }} \mathop \sum \limits_{j = 1}^{{N_{g} }} \left| {\left. {i - j} \right|} \right..p\left( {i,j} \right)$
Entropy^78^		$- \mathop \sum \limits_{i = 1}^{{N_{g} }} \mathop \sum \limits_{j = 1}^{{N_{g} }} p\left( {i.j} \right)log p\left( {i,j} \right)$
Energy^78^		$\mathop \sum \limits_{i = 1}^{{N_{g} }} \mathop \sum \limits_{j = 1}^{{N_{g} }} p\left( {i - j} \right)^{2}$
Homogeneity^84^		$\mathop \sum \limits_{i = 1}^{{N_{g} }} \mathop \sum \limits_{j = 1}^{{N_{g} }} \frac{{p\left( {i,j} \right)}}{{1 + \left( {i - j} \right)^{2} }}$
Maximum probability^84^		$$ max_{ij } p(i,j$$)
Sum average^78^		$\mathop \sum \limits_{k = 2}^{{2N_{g} }} k p_{x + y} \left( k \right)$
Sum entropy^78^		$- \mathop \sum \limits_{k = 2}^{{2N_{g} }} p_{x + y} \left( k \right)\log p_{x + y} \left( k \right)$
Inverse difference^85^		$\mathop \sum \limits_{i = 1}^{{N_{g} }} .\mathop \sum \limits_{j = 1}^{{N_{g} }} \frac{{p\left( {i,j} \right)}}{{1 + \left| {i - j} \right|}}$
Information measure of correlation 1^78^		$\frac{HXY - HXY1}{{{\max}\left( {HX,HY} \right)}}$
Information measure of correlation 2^78^		$\sqrt {1 - {\exp}\left[ { - 2\left( {HXY2 - HXY} \right)} \right]}$

Where: $p\left( {i,j} \right)$ is the ith and jth entry in the normalized gray level dependence matrix.

$$p_{x} \left( i \right) = ith\; entry\; in\; probability\; matrix,\; p_{x} \left( i \right) = { }jth\; entry\; in\; probability\; matrix.$$

$$N_{g = } no\,of\,gray\,scales, {\varvec{p}}_{{\varvec{x}}} \left( {\varvec{i}} \right) = \mathop \sum \limits_{j = 1}^{{N_{g} }} p\left( {i,j} \right),{ } \user2{ p}_{{\varvec{y}}} \left( {\varvec{j}} \right) = \mathop \sum \limits_{i = 1}^{{N_{g} }} p\left( {i,j} \right),{ }{\varvec{p}}_{{{\varvec{x}} + {\varvec{y}}}} \left( {\varvec{k}} \right) = { }\mathop \sum \limits_{i = 1}^{{N_{g} }} { }\mathop \sum \limits_{j = 1}^{{N_{g} }} p\left( {i,j} \right){ }; i + j = k$$

$${\varvec{p}}_{{{\varvec{x}} - {\varvec{y}}}} \left( {\varvec{k}} \right) = { }\mathop \sum \limits_{i = 1}^{{N_{g} }} { }\mathop \sum \limits_{j = 1}^{{N_{g} }} p\left( {i,j} \right){ };\left| {i - j} \right| = k,$$$${\varvec{HX}} = - \mathop \sum \limits_{i = 1}^{{N_{g} }} p_{x} \left( i \right).logp_{x} \left( i \right), {\varvec{HY}} = - \mathop \sum \limits_{i = 1}^{{N_{g} }} p_{y} \left( i \right).logp_{y} \left( i \right),{ } {\varvec{HXY}} = - \mathop \sum \limits_{i = 1}^{{N_{g} }} { }\mathop \sum \limits_{j = 1}^{{N_{g} }} p\left( {i,j} \right).\log p\left( {i,j} \right)$$

$${\varvec{HXY}}1 = - \mathop \sum \limits_{i = 1}^{{N_{g} }} { }\mathop \sum \limits_{j = 1}^{{N_{g} }} p\left( {i,j} \right).log\left[ {p_{x} \left( i \right).p_{y} \left( j \right)} \right]$$

####  Feature selection

Feature selection removes extraneous features, leading to improved model prediction. In the present study, ANOVA was used to extract the best features for classifying images; this applies statistical measures to assign scores to each feature, retaining the top-ranked features in the system and removing the low-ranked features. The top-ranked five features were selected and fed to the classifier for further processing.

#### Classification

The texture features of the IR eye-fundus images were classified into two groups using an SVM: normal and ME eyes. SVMs are reliable and practical classifiers for small datasets, can be applied in classifications and regression analyses and have been previously used for similar applications^42,44,86^. A linear function was used in this model, and the dataset was divided into training (50%) and test sets (50%).
